# Age-related differences in skeletal muscle microvascular response to exercise as detected by contrast-enhanced ultrasound (CEUS)

**DOI:** 10.1371/journal.pone.0172771

**Published:** 2017-03-08

**Authors:** Wulf Hildebrandt, Hans Schwarzbach, Anita Pardun, Lena Hannemann, Björn Bogs, Alexander M. König, Andreas H. Mahnken, Olaf Hildebrandt, Ulrich Koehler, Ralf Kinscherf

**Affiliations:** 1 Department of Medical Cell Biology, Institute of Anatomy and Cell Biology, University of Marburg, Marburg, Germany; 2 Department of Diagnostic and Interventional Radiology, University Hospital of Giessen and Marburg (UKGM) University, Baldingerstraße, Marburg, Germany; 3 Department of Sleep Medicine, Division of Pneumology, Internal Medicine, University Hospital of Giessen and Marburg (UKGM) Baldingerstraße, Marburg, Germany; Augusta University Medical College of Georgia, UNITED STATES

## Abstract

**Background:**

Aging involves reductions in exercise total limb blood flow and exercise capacity. We hypothesized that this may involve early age-related impairments of skeletal muscle microvascular responsiveness as previously reported for insulin but not for exercise stimuli in humans.

**Methods:**

Using an isometric exercise model, we studied the effect of age on contrast-enhanced ultrasound (CEUS) parameters, i.e. microvascular blood volume (MBV), flow velocity (MFV) and blood flow (MBF) calculated from replenishment of Sonovue contrast-agent microbubbles after their destruction. CEUS was applied to the vastus lateralis (VLat) and intermedius (VInt) muscle in 15 middle-aged (MA, 43.6±1.5 years) and 11 young (YG, 24.1±0.6 years) healthy males before, during, and after 2 min of isometric knee extension at 15% of peak torque (PT). In addition, total leg blood flow as recorded by femoral artery Doppler-flow. Moreover, fiber-type-specific and overall capillarisation as well as fiber composition were additionally assessed in Vlat biopsies obtained from CEUS site. MA and YG had similar quadriceps muscle MRT-volume or PT and maximal oxygen uptake as well as a normal cardiovascular risk factors and intima-media-thickness.

**Results:**

During isometric exercise MA compared to YG reached significantly lower levels in MFV (0.123±0.016 vs. 0.208±0.036 a.u.) and MBF (0.007±0.001 vs. 0.012±0.002 a.u.). In the VInt the (post-occlusive hyperemia) post-exercise peaks in MBV and MBF were significantly lower in MA vs. YG. Capillary density, capillary fiber contacts and femoral artery Doppler were similar between MA and YG.

**Conclusions:**

In the absence of significant age-related reductions in capillarisation, total leg blood flow or muscle mass, healthy middle-aged males reveal impaired skeletal muscle microcirculatory responses to isometric exercise. Whether this limits isometric muscle performance remains to be assessed.

## Introduction

As an important manifestation of age, the response of (sub)maximal limb blood flow or vascular conductance to dynamic exercise declines in humans [1–6] and in mammals [7, 8]. Though still equivocal for the forearm [3, 9, 10], this finding is valid for human leg muscles and likely contributes to age-related decreases in aerobic capacity and arterio-venous oxygen difference across in the leg [11, 12]. Impairment in exercise blood flow appears to start as early as from the age of ~40 years, at least in males, and has been suggested as one out of several other factors of age-related losses in muscle function and mass which become manifest at similar age [13–16]. Importantly, these age-related limitations of exercise blood flow are not due to decreased limb muscle mass itself [4, 17] or to differences in metabolism, because they remain detectable even upon passive limb movement [18, 19]. Of note, leg exercise training effectively reverses age-related reductions in peripheral O_2_-availability in dynamically exercising leg muscle [2, 6, 12, 20, 21].

These age-related limitations in exercise blood flow may be attributable to *structural* as well as *functional* vascular factors, which are mostly training-sensitive: As a *structural* factor, there clearly is a loss in capillary density or fiber contacts with age [22, 23] despite a largely preserved angiogenetic exercise response [24]. Such capillary rarefaction may limit maximal vascular conductance as well as the size (exchange surface) of the fiber adjacent capillary network within a single microvascular unit (MVU), which upon exercise is considered recruite fiber-adjacent exchange surface by several mechanisms (see Discussion) at increased red blood cell (rbc) and/or plasma flux depending on proximal vasomotion of terminal arterioles or, if at all present, pre-capillary sphincters, though human data on the microvascular architecture are still lacking [25]. Beside age and sedentary life style, the metabolic syndrome as an age-immanent factor itself may contribute to capillary rarefaction [26]. Furthermore, aging may lead to remodeling of proximal feed arteries resulting e.g. increased femoral intima-media thickness (IMT) and IMT/lumen ratio [21, 27, 28].

Regarding *functional* factors, age-related impairement in local vasodilation in exercising muscle may occur predominantly through endothelial dysfunction [3, 29]. Endothelial function at rest in terms of flow-mediated (post-occlusive) or acetylcholine-induced vasodilation, clearly decays from the age of 40 in men and 50 in women (i.e. in line with gender-specific cardiovascular risks) while, in contrast, endothelial-independent vasodilation via glyceryl trinitrate is unaffected by age [16]. Among further possible factors of limited exercise hyperemia with ageing [3], sympathetic nerve activity, though increased and related to blood flow at rest in elderly [18], was recently excluded, at least in the forearm [17].

However, there presently is a need for microcirculatory studies regarding age-related exercise response in humans, because flow distribution by recruitment of capillary exchange surface within MVU during exercise, may not be reflected by *total* limb blood flow recordings. In fact, local vasomotion factors implicated in exercise like pO_2_ appear to differentially impact the reactivity among branches of microvascular resistance networks, as demonstrated in mammals [30]. There are indeed several previous microvascular studies using contrast enhanced ultrasound (CEUS) in human skeletal muscle, that report an age-related attenuation of microvascular response to insulin (as another important physiological stimulus beside exercise) which was not detected by total limb blood flow [31–34]. Similar results were obtained for metabolic syndrome and type-2-diabetes as age-related conditions [35–39], supporting a novel ‘vascular paradigm’ of insulin resistance, i.e. an attenuation of insulin-mediated recruitment of capillary exchange surface and transendothelial insulin transport as rate limiting steps for insulin (and glucose) delivery to skeletal muscle [36, 39–45]. Regarding exercise-induced microvascular response, human studies are limited to patients with diabetic microangiopathy [46]. The present study therefore evaluated i) age-related alterations in microvascular exercise response of skeletal muscle using CEUS and, in addition, ii) the capillary histomorphometry in biopsies from the actual CEUS site, because capillarisation is an important structural factors within ageing or the metabolic syndrome not addressed by previous human CEUS studies [22, 23, 26, 39, 47–49].

We hypothesized, that—in line with endothelial function [16]—microvascular exercise response would be compromised in males within the 5^th^ decade, but not necessarily explained by lower capillary density alone or reflected by total limb blood flow as indicated in [31, 33].

The little-invasive *in-vivo* method of CEUS has been demonstrated to assess both, exercise- and insulin-induced microvascular responses in humans and in mice [33, 49, 50] by detecting circulating contrast-agent microbubbles at low mechanical index (MI) during their real-time replenishment immediately after their high-MI destruction in intramuscular region of interest (ROI). Under equilibrated conditions i.e. a stable arterial inflow microbubble concentration, replenishment curves (RC) can be modelled using y = y0 + α(1- e^-βt^) [51], which yields microvascular blood volume (MBV, α) within the ROI (when excluding larger vessels), microvascular flow velocity (MFV, β) and the microvascular blood flow (MBF, α*β), i.e. the initial slope of the RC. Isometric contraction was presently chosen as an exercise model, because dynamic exercise involves instable conditions regarding intra- or transmural pressures, metabolism, or blood flow, largely excluding CEUS RC recordings of up to 30s. Moreover, light isometric exercise, e.g. 15% of peak torque (PT), can be individually well defined and tolerated for several minutes as required for CEUS [52].

We presently show rather early impairements in microcirculation during or after prolonged isometric contraction of the vastus lateralis (VLat) and intermedius (VInt) muscle in middle-aged (MA) compared to young (YG) healthy males. These limitations are not well reflected by femoral artery blood flow and not due to capillary rarefication. Importantly, MA and YG had very similar quadriceps muscle volume or strength and maximal oxygen uptake and, moreover, normal intima-media thickness (IMT) and vascular risk factors [26, 53, 54].

## Methods

### Subjects and study design

**Fig 1:**
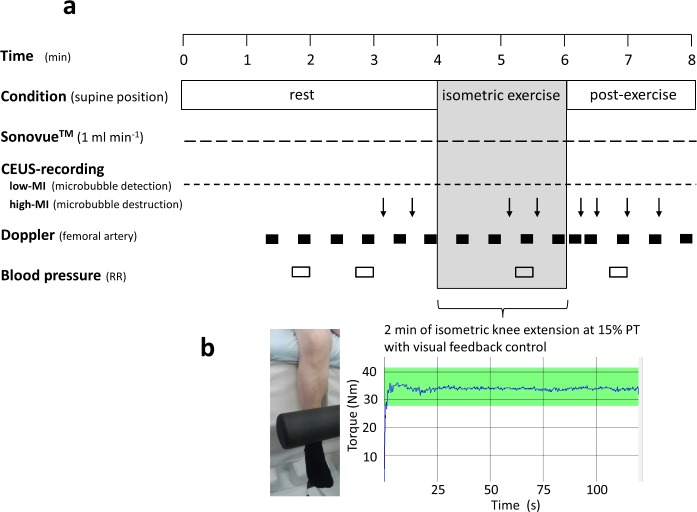
‘Experimental protocol’. Fig 1a) Study protocol with time schedule for exercise, Sonovue infusion, CEUS recordings, as well as femoral artery Doppler and brachial blood pressure measurements at rest, during isometric exercise and during post-exercise hyperemia. The time points of high-MI US-destruction of the Sonovue microbubbles are indicated by arrows, each of which was followed by a low-MI recording of Sonovue replenishment curves covering 25 s. Fig 1b) Example of a torque recording during isometric knee extension as controlled by the subject through visual feed-back.

The present cross-sectional study evaluated age-related differences of CEUS-parameters within both the VLat and VInt muscle at rest and in response to 2 min of isometric knee extension at the work load of 15% of peak torque (PT), which significantly increases femoral arterial i.e. total leg blood flow (see **Fig 1 ‘Experimental protocol’** Fig 1A and 1B).

26 normotensive healthy volunteers, i.e. 15 middle-aged (MA, aged 36–56 years) and 11 young (YG, age 20–28 years) males with reported regular physical activity were recruited to the study by public announcement. All subjects underwent an initial medical examination including their medical history and physical activity profile, a physical examination, routine laboratory venous blood parameters, bilateral blood pressure measurement in sitting position, pulmonary function, and a 12-lead electrocardiogram (ECG) at rest. Screening for vascular risks furthermore included routine measurement of right and left intima-media-thickness (IMT) at the arteria carotis communis. In addition, the subjects’ exercise capacity and maximal oxygen uptake (VO_2_max) were assessed by an incremental cycle ergometer test using the Ergoline 100 system (Ergoline, Windhagen, Germany) with work load increments of 25 W every 2 min starting from 50 W until exhaustion. Thereby, using the ZAN 680 system (ZAN Ferraris Cardiorespiratory, Oberthulba, Germany), breath-by-breath spirometry data including inspiratory and end-tidal gas fractions, furthermore a 12-lead ECG, and left index finger tip oxymetry data (Nonin 7500, nSpire Health, Oberthulpa, Germany) were continuously recorded and stored together with automated left brachial blood pressure measurements taken at each work load. VO_2_max was calculated as the maximum of mean values covering intervals of 10 s.

Exclusion criteria were: VO_2_max <30 ml kg^-2^, vascular risks as indicated by IMT >0.8 mm, supra-normal plasma levels of LDL, total cholesterol, furthermore any medication or supplementation of medication including C- or B-vitamins/folate; body mass index (BMI) <19 or >33 kg m^-2^, hypertension (RR systolic >145 mmHg, diastolic >100 mmHg, hypotension (RR systolic <90 mmHg), any history of or cycle ergometer test-related symptoms of cardiovascular/-respiratory disease or events, any other major intestinal, hepatic, renal, musculoskeletal, dermatological or neuropsychiatric clinical condition; any present or previous (<6 months before inclusion) blood donation or high altitude sojourne above 2000 m altitude; any alcohol or drug abuse; missing oral or written consent; insufficient cooperation or poor tolerance to the exercise protocol; any known intolerance to or symptoms related to the CEUS contrast agent Sonovue. Any reported susceptibility to keloid (with regard to skin incision for muscle biopsy).

Approval was obtained from the local Ethical Committee of the University Hospital of Marburg (ethikkom@staff.uni-marburg.de, ID number: 63/11, 12.09. 2011) and the study performed according to the amended Declaration of Helsinki (1996). All volunteers were enrolled after their informed oral and written consent was obtained.

### Blood sampling

Upon arrival of the fasted subjects at the laboratory between 8:00 and 10:00 a.m., postabsorptive blood samples were drawn from an antecubital vein after 15 min rest and processed for clinical routine laboratory diagnostic in the central laboratory of the University Hospital of Gießen and Marburg (UKGM) included plasma levels triglycerides, total cholesterol, very-low-density-lipoprotein (VLDL), low-density-lipoprotein (LDL), high-density lipoprotein (HDL), homocysteine, fasted glucose, HbA1c, as well as a differential hemogram, hemoglobin, and hematocrit, furthermore routine laboratory screening parameters for liver, renal and muscle diseases.

### Contrast enhanced US (CEUS)

#### Apparative setting and procedure

**Fig 2:**
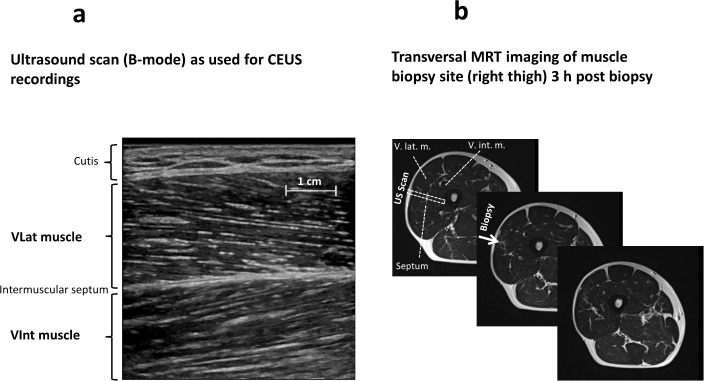
‘Ultrasound and MRT imaging of CEUS and Biopsy muscle site’. Fig 2 a) US B-mode image of a typical combined vastus lateralis (VLat) and intermedius (VInt) CEUS scan with the scanner position chosen parallel to the VLat muscle fiber orientation (i.e. from proximal/lateral to distal/medial). The intramuscular septum separating both muscles is indicated, the mean depth was similar between middle aged (MA) and young (YG) subjects and not significantly different between the conditions before, during, and post-exercise (see also the ‘Methods‘ section on Contrast-enhances US (CEUS). d) Thigh MRT-imaging transversal section at the exact site of VLat muscle biopsy (*middle*) and CEUS recording as well as 1 cm proximal (*left*) and distal (*right*). Note that this MRT was obtained 3 h after a muscle biopsy to visualize the exact biopsy site (local fluid /blood accumulation) indicated by the arrow.

CEUS was performed using the Logiq E9 system (General Electrics GE, Connecticat, USA; software version R3.1.2) in combination with the 9D-L linear scanner. Sonovue microbubbles in the VLat and VInt muscle were detected by use of the Logiq E9 ‘Contrast’ mode (‘Resolution’) with the following settings: Mechanical index (MI) 0.11, acoustic output (AO) 7%, gain 14%, dynamic range 60 dB, and frame rate ~20 s^-1^. High-MI-flashs for microbubble destruction were applied at 100% AO (MI 1.2) with 7 frames covering ~0.4 s. The 9D-L scan area was set to 4.5 cm depth at 4 cm width and the focus fixed at 3.2 cm. The depth of the intramuscular septum between the VLat and VInt muscle at rest, during exercise, and post-exercise was 3.21±0.15, 3.29±0.14, and 3.13±0.17 cm in MA and 3.39±0.11, 3.48±0.09, and 3.34±0.09 cm in YG, respectively. An example of a B-mode US-image of the area scanned is given in **Fig 2 ‘Ultrasound and MRT imaging of CEUS and Biopsy muscle site’ (**Fig 2A). Moreover, the localisation of the US B-mode or CEUS Contrast-mode scan, including the actual site of muscle biopsy (indicated by an arrow), is shown in Fig 2B (transversal MRT image). (See also ‘**S1 Video. Example of a CEUS recording at rest, during and after isometric contraction’** of the real-time and the cumulative CEUS recordings at rest, during exercise and post-exercise).

The 9D-L scanner was angled in parallel to VLat fiber orientation and placed at about the VLat maximal convexity with experimental isometric contraction at 15%PT, i.e. between the middle and distal third of the thigh. The position and skin contact of the scanner was secured by special foamed plastic support excluding pressure exertion to the insonated tissue. Upon antecubital Sonovue infusion at a rate of 1 ml min^-1^ the low-MI CEUS-signal was continuously recorded covering an 8 min interval including several replenishment curves of 25 s after high-MI microbubble destruction under conditions of rest, 2 min of isometric contraction and 2 min post-exercise conditions (see Fig 1A and **‘S1 Video. Example of a CEUS recording at rest, during and after isometric contraction’**).

**Fig 3:**
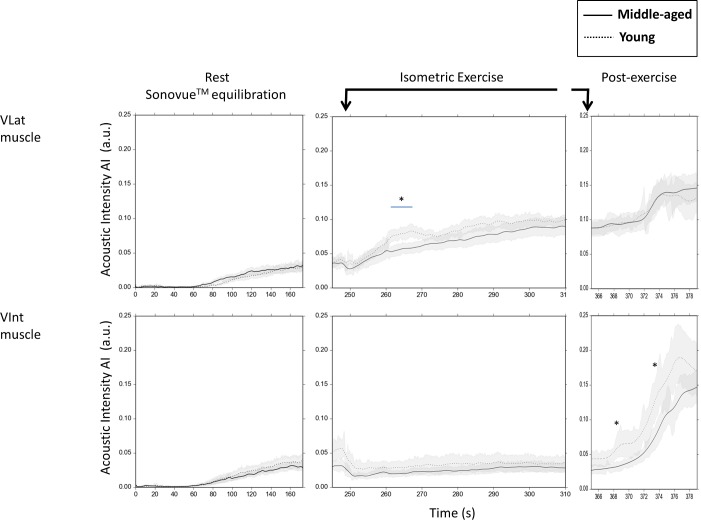
‘Time courses of the mean contrast-agent CEUS signals’. Time course of the mean (±SEM) Sonovue microbubble signal in the vastus lateralis (VLat; *upper panel*) and the vastus intermedius (VInt; *lower panel*) muscle region of interests (ROI) in middle-aged (MA, n = 15) and young (YG, n = 11) males in the experimental intervals: equilibration at rest (*left;* initial 180 s of Sonovue infusion), isometric exercise (*middle*; first 60 s of knee extension at 15% of PT), and post-exercise (*right;* initial 15 s after cessation of). Note the different time scales on the x-axis with these three conditions. The time intervals for repetitive Sonovue replenishment curve (RC) recording following high-MI US destruction of Sonovue microbubbles (See Fig 1) are presented separately in Fig 4A (mean RC curves) and Fig 4B (means of the individual regression lines obtained from individual RC curves). * for p<0.05 MA vs YG by unpaired Student’s t-test.

In detail, the initial 30 s of CEUS following the start of Sonovue infusion were used as (microbubble-free) background for CEUS calculations explained below (see **Fig 3 ‘Time courses of the mean contrast-agent CEUS signals’** left panel).

Out of the initial 240 s of resting period, the initial 180 s allowed for complete intravascular Sonovue equilibration (Fig 3, left panel) followed by two consecutive replenishment curve recordings (covering 25 s each) for duplicate CEUS analyses at rest. During the following 2 min of knee extension at 15% PT, the initial 70 s were observed without high-MI microbubble destruction and, thereafter, two consecutive 25-s-RC were obtained within the last 50 s of contraction. Upon exercise cessation, i.e. during a post- exercise hyperemic interval, CEUS-RCs were recorded after 15 s (RC of 15 s only) and after 30, 60, and 90 s (each covering >25 s). Arterial pressure readings were obtained at time points indicated in Fig 1A using the automated device Visomat Comfort 20/40 (Uebe, Wertheim, Germany).

#### Contrast agent Sonovue

The commercially available contrast agent Sonovue (Bracco Imaging, Konstanz, Germany) consisting of coated SF_6_-containing microbubbles was prepared according to the manufacturer’s instruction using two 4.8 ml vials per subject. The resulting total Sonovue amount recovered in a 10ml syringe ranged between 8.5–8.8 ml and was infused through an antecubital vein at a constant rate of 1 ml min^-1^ using the rotating Viewject pump system (Bracco, Milano, Italy) including the volume-adjusted infusion system provided by this manufacturer. Thus, an overall experimental protocol of 8-min was covered by constant Sonovue infusion allowing for CEUS analyses before, during, and after exercise as given in Fig 1A. For initial intravascular Sonovue equilibration an interval of 3 min was considered sufficient (Fig 3, left panel) and in line with two previous studies on CEUS methodology [33, 50].

#### CEUS calculations

**Fig 4:**
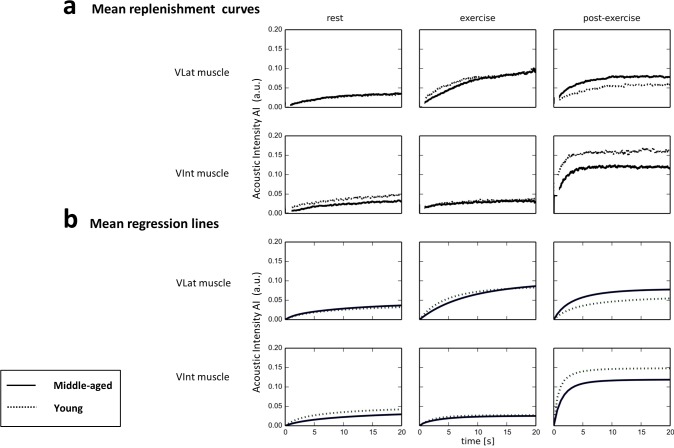
‘Means of measured replenishment curves and individual regression lines’. Fig 4a) Mean replenishment curves (RC) in the vastus lateralis (VLat; *upper panel*) and the vastus intemedius (VInt; *lower panel*) muscle ROI of middle-aged (MA, n = 15) compared to young (YG, n = 11) males during rest (left), after 70 s isometric exercise (middle), and 15 s post-exercise. Note differences in initial slope or in the plateau reached during or post-exercise. Fig 4b) Mean regression lines, corresponding to mean RC presented above in a) i.e. for the VLat (*upper panel*) and the VInt (*lower panel*) muscle ROI of middle-aged (MA, n = 15) compared to young (YG, n = 11) males during rest (left), after 70 s isometric exercise (middle), and 15 s post-exercise. Note the differences in initial slope or reached plateau during or post exercise. Furthermore, note that regression lines represent the mean of individual regression lines calculated for individual RCs (not the regression line calculated for mean RCs, presented above in a). For statistical differences between MA and YG regarding the RC-derived parameters of microvascular blood volume (MBV), flow velocity (MFV), and blood flow (MBF) please see Fig 5.

Replenishment curves (RC) were obtained from exported DICOM format cine-loops of 2 min length using the non-log-compressed acoustic intensity (AI) signal. The post-flash RC at rest, during, and after exercise (see **Fig 4 ‘Means of measured replenishment curves and individual regression lines’**, Fig 4A) were modelled individually by non-linear regression (Fig 4B) according to Wei et al. [51], i.e. via the equation: acoustic intensity y = y0 + α(1- e^-βt^), with t = given time, α = plateau (microvascular blood volume, MBV), β = time constant (microvascular flow velocity, MFV) and α x β = initial slope (microvascular blood flow, MBF). The regression was based on AI data between 1 s and 25 s post-flash. The y-value as well as x-value of the post-flash ‘origin’ of the RC was determined by two methods:

1. by calculating the y-value (AI) at the time point of the last high-MI video frame (when microbubble tissue concentration must be minimal) by extrapolation of the regression line (determined by regression for RC data between 1 s and 25 s post-flash) (see S1A Fig ‘new’ in ‘S1 Fig. Methodology of CEUS replenishment curve calculation’,). This is a slightly modified, new approach compared to
2. the hitherto commonly used method [33, 50], i.e. calculating the RC origin as the mean of AI values during the first ~0.5 s post flash (see also S1A Fig, ‘conventional’ in ‘S1 Fig. Methodology of CEUS replenishment curve calculation’).

This present method a) was chosen for presentation of results, because the RC origin definition massively impacts both α and β and thus especially their product (MBF) but may be easily confounded (see **S1B Fig. Methodology of CEUS replenishment curve calculation’**). In fact, RC origin as determined commonly by method b) appears to vary quite widely from the background signal (without microbubbles), which implies that calculated α (MBV) may not represent well and mostly considerably underestimate the background-subtracted microbubble signal obtained immediately before the high-MI flash, as already indicated (see Fig 3C in [33]). This might putatively reflect confounders like early microbubble inflow within the 1^st^ s (not necessarily only in large vessels), incomplete high-MI microbubble destruction within the ROI, partial microbubble destruction outside the ROI margin affecting the (initial) RC of the scanned/flashed tissue area, moreover, pulsatile microvascular flow (especially at increased blood flow) and other factors including apparative delay in switching from high-MI-destruction to post-flash low-MI CEUS recordings. Therefore, the present method a) used the power the RC curve between 1s - 25s to define the y-minimum (RC origin) at the end of the flash, i.e. the point of time closest to an actual AI minimum (probably not detected by the US devices for CEUS).

For comparison of the two methods see S1B **Fig** **in ‘S1 Fig. Methodology of CEUS replenishment curve calculation’**, which presents method a) (‘new’) and b) (‘conventional’) in terms of the percentage loss of background-subtracted microbubble signal upon high-MI microbubble destruction. Obviously, the present method a) (‘new’) quite well represents a 100% loss of background-subtracted microbubble signal at RP origin, while the method b) (‘conventional’) appears to generally underestimate the microbubble signal loss at RP origin, especially with very low microvascular blood flow of the VInt during exercise (**‘S1 Fig. Methodology of CEUS replenishment curve calculation’**,).

#### CEUS ROI selection

For separate regional CEUS analyses of the VLat and VInt, the video clip images saved as PNG and calibrated with a Gamma value of 2.2 (see **‘S1 Video. Example of a CEUS recording at rest, during and after isometric contraction’**) were subdivided into 16 slices of 2.5mm height for quantification of AI normalized for maximum of the echo value domain. For defining representative and interindividually comparable ROIs, a serial of slices between 19–28 mm depth and between 39-45mm depth was selected because it represented well the VLat and the VInt muscle in all subjects, respectively, without interference of the vessel-rich intramuscular septum at both rest and exercise. For each of these two ROIs the RCs were calculated according to the above mentioned model equation using the above mentioned time window between 1 s and 25 s after high-MI microbubble destruction.

### Duplex-Doppler-flow measurement

Total leg blood flow was assessed by the Duplex-Doppler-flow measurement from the proximal femoral artery with the above- mentioned US system (Logiq E9 with 9D-L scanner, Doppler mode) together brachial arterial blood pressure readings according to the study protocol given in Fig 1A. A velocity-time-integral (VTI) was obtained covering about 12 s per each blood flow measurement every 30 s during the last 3 min or baseline rest (lasting at least 30 min in total), during 2 min of isometric knee extension, and for 5 min thereafter. Femoral artery inner diameter, assessed as the mean of 4 measurements, was used to calculate the luminal cross-sectional area (CSA) for calculation of Doppler flow in terms of the product of VTI x CSA normalized for time (ml min^-1^). The recording of femoral artery Doppler-flow and that of VLat or VInt muscle CEUS were obtained on the same day by two identical exercise protocol sessions which were randomized and separated by at ~60 min rest. Total leg vascular conductance was determined as Doppler (in)flow per mean arterial pressure, which was calculated as *diastolic pressure + (systolic pressure—diastolic pressure) /3* with correction for hydrostatic differences to the distal thigh, assuming venous outflow pressure to be near zero (which is realistic when placing the relevant quadriceps muscle portion above heart level as presently done).

### Resting calf blood flow and arterial flow reserve

Both these parameters were bilaterally assessed after >30 min supine rest by means of venous occlusion plethysmography using the automated, calibrated (linked-chain) strain gauge system Compactus 2000 (Sogut GmbH, Geretsried, Germany). Thereby the strain gauge was mounted at around maximal calf circumference placed ~15 cm above heart level and the venous/arterial occlusion cuff placed around the proximal thigh with padded leg support rendered at the distal thigh and heels. Six resting calf blood flow values were obtained within 90 s using the linear initial slope of volume increase upon 6 s of venous occlusion at 60 mmHg rapidly reached and regulated by the automated device through a pressure reservoir. For calf maximal flow reserve, the thigh occlusion cuff was inflated to 240 mmHg for 3 min (automated control) and immediate post-occlusion peak blood flow determined out of 4 blood flow readings every 15 s. Three min instead of the more common 5 min arterial occlusion time was chosen to reduce discomfort or pain caused by ischemia and/or cuff pressure.

### Muscle mass and function

#### Quadriceps muscle MRT-volugraphy

Muscle mass was quantified in terms of the quadriceps muscle total volume between the trochanter minor and the patella by a 1.5 T whole-body MR scanner (Siemens Espree, Erlangen, Germany), equipped with a body matrix and a peripheral angio coil. The images were acquired using a T2 turbo-spin-echo (TSE) sequence with following parameters: TR = 5490ms, TE = 91ms, number of averages (NEX) = 3, slice thickness = 4.0mm, base resolution = 256, interslice gap = 0% and composing algorithm: spine (syngo MR B17). The total acquisition time was 13.4 min. For the volugraphy of all volunteers the software OSIRIX (OSIRIX v.6.0.2 32-bit, Pixmeo, Bernex, Switzerland) was used.

#### Maximal handgrip strength

This parameter was bilaterally determined using the handgrip dynamometer (Baseline HD Hydraulic Dynamometer, Cando, Osaka, Japan) via five consecutive trials performed in standing position with dependent arms and interspersed breaks of 3 min.

#### Knee extension peak torque and isometric test workload

Right and left voluntary maximal PT of isometric knee extension was determined as the maximum of 5 trials lasting 3–5 s each and separated by breaks of at least 3 min using the M3-Diagnos professional system (Schnell, Peutenhausen, Germany). Measurements were performed in a comfortable supine position at 30° and 90° flexion of the hip and the knee, respectively, which was identical to the actual CEUS or Doppler study position. For the 2 min intra-exercise recordings of both CEUS and femoral artery US-Doppler (see Fig 1A), the individually isometric knee extension workload was defined as 15% of the individual PT which was determined at least 24 h beforehand. Maintenance of constant isometric workload at 15% PT for 2 min was controlled by visual feedback (see example in Fig 1B), with this procedure being practiced several times at least 24 h before the study day.

### Muscle biopsies

#### Sample taking and processing

Muscle biopsies were obtained from 13 out of 15 MA and 10 out of 11 YG subjects from the VLat muscle using a conchotome of 6 mm width through an 8 mm skin and fascia incision after careful local anesthesia and desinfection. Exact biopsy localisation within the VLat was controlled by MRT 2 h after sampling (Fig 2B). Muscle samples were immediately shock-frozen in liquid nitrogen-cooled isopentane and stored at -80°C. Serial transverse cryo-sections (6–7 μm) were cut in a cryostat microtome (Hyrax C60, Carl Zeiss AG, Oberkochen) at -20°C for histomorphometry of capillarisation and fiber composition.

#### Fiber typing by adenosine triphosphatase (ATPase) staining

For fiber type 1, 2a, or 2x identification, cryo-sections were histochemically stained for acid-sensitive myofibrillar ATPase (Adenosine triphosphatase, Sigma-Aldrich Co. LLC, St. Louis, Missouri) after preincubation at pH 4.6 (5 min, room temperature) as previously described [55]. Rectangular areas of intact tissue chosen for analysis of fiber composition (percentage of type 1, 2a and 2x fibers) comprised >100 fibers with a comparable mean fiber count. Mean overall and type-specific fiber cross-sectional areas were determined in images taken at a 200-fold magnification by the Zeiss Axio Imager.M2 microscope (Carl Zeiss AG; Oberkochen, Germany) after digitalization by the digital high-resolution imaging system Axio-Cam HRc/AxioVision (Carl Zeiss GmbH) using standard imaging software ImageJ (Scion Image, National Institutes of Health, Bethesda, USA).

#### Immunohistochemistry of capillary staining

For visualization of capillaries, directly neighboured transverse sections were selected, fixed in 4% PFA/ PBS 10 min treated with 3% H_2_O_2_ for endogenous peroxidase blockage as well as with 1% normal porcine serum in PBS for blockage of non-specific sites. Sections were then incubated 30 min at 37°C with the mouse anti-human CD31 primary antibody (Dako Deutschland GmbH, Hamburg, Germany) which was detected using the horseradish-conjugated anti-mouse secondary antibody system (Vectastatin ABC Kit, Vector Laboratories TTD, Peterborough, U.K.) via chromogen 3–3’-diaminobenzidine-tetrahydrochlorid (DAB, Roche Diagnostics, Mannheim, Germany) at room temperature. Nuclei were counterstained with Mayer's hematoxylin (Carl Roth GmbH, Karlsruhe, Germany) and sections cover-slipped in mounting medium for histomorphometry (DePeX, SERVA Electrophoresis GmbH, Heidelberg, Germany). Histomorphometric analyses of directly muscle fiber-adjacent capillarisation (capillaries of ~5 μm diameter) at 200x magnification comprised capillary density (CD, count per mm^2^ rectangle area analysed) and capillary count per total fiber area within the rectangle analysed. Moreover, the overall fiber capillary contacts as well as fiber type-specific capillary contacts (including shared capillaries) were determined by relating each capillary contact to its single type 1, 2a, or 2x fiber in a directly neighboured ATPase-stained section. Furthermore, the capillary contacts of all and of type 1, 2a, or 2x fibers per cross sectional fiber area was calculated as an inverse index of nutritive capillary supply.

### Statistics

Results were presented as means ± S.E.M. for the MA and the YG group separately. For cross-sectional comparison between both groups, the unpaired Student’s t-test or, whenever adequate, the Man-Whitney U test was used. Exercise-related intraindividual changes were tested within each group by the Student’s t-test for paired observation. A P-level <0.05 was considered as statistically significant. The SPSS-software version 22.0 (IBM, Munich, IBM, Munich, Germany) was used for all statistical procedures.

## Results

### Comparability in anthropometric, cardiovascular risks and muscle functional parameter between MA and YG

The anthropometric data (**Table 1: ‘Anthropometry, cardiovascular risk factors as well as leg muscle mass, function and vascularisation’**) show that, despite their about 20 years higher age compared to YG, MA had similar body height and muscle mass or function as assessed by handgrip maximal strength, quadriceps (knee extensor) isometric PT and MRT-volume. Furthermore, their exercise capacity/fitness was within the same range as that of YG, as reflected by resting heart rate and VO_2_max (l min^-1^). This comparability with regard to muscle mass and function was achieved at the cost of a significantly 10 kg higher body weight and accordingly a 3.8 kg m^-2^ higher BMI in MA compared to YG, which most likely reflects increased absolute and percentage body fat. Such age-related change in nutritional /metabolic state, i.e. body fat-related higher BMI, may be considered to represent ‘normal healthy aging’ in Western industrial countries. This reading may also be reflected by the concomitant marginal (but in case of diastolic values significant) increase in brachial arterial blood pressure, which, however, remained well within the normotensive range.

**Table 1:** Anthropometry, cardiovascular risk factors as well as leg muscle mass, function and vascularisation.

			Young (YG)	Middle-aged (MA)
N			11	15
Age	(years)		24.1 ±0.7	43.6 ±1.5***
Body weight	(kg)		78.6 ±2.1	88.7 ±2.7*
Body height	(cm)		183.9 ±1.6	182.2 ±1.4
Body mass index	(kg m^-2^)		23.1 ±0.8	26.9 ±0.8**
Triglycerides	(mg 100ml^-1^)		67.7 ±10.7	108.4 ±15.2
Total cholesterol	(mg 100ml^-1^)		156.3 ±8.3	210.3 ±8.8***
LDL	(mg 100ml^-1^)		98.5 ±7.7	144.1 ±7.2**
HDL	(mg 100ml^-1^)		49.5 ±3.2	49.5 ±2.8
Homocysteine	(μg ml^-1^)		11.3 ±2.3	11.9 ±1.9
Glucose (fasted)	(mg 100ml^-1^)		89.3 ±1.6	93.5 ±1.9
HbA1c	(%)		5.20 ±0.07	5.45 ±0.07*
Heart rate (supine)	(1 min^-1^)		64.2 ±2.9	69.0 ±2.7
RR systolic	(mmHg)		127.8 ±2.2	132.8 ±1.5
RR diastolic	(mmHg)		75.4 ±3.0	85.8 ±1.7*
Carotid IMT	(mm)	left	0.51 ±0.03	0.62 ±0.03*
		right	0.51 ±0.03	0.60 ±0.03*
Handgrip strength	(kg)	left	55.1 ±2.5	55.6 ±2.1
		right	58.5 ±2.1	58.8 ±2.6
Knee extension PT	(Nm)	left	231.2 ±16.8	225.9 ±14.7
		right	217.3 ±16.5	228.3 ±13.9
Quadriceps muscle volume	(cm^3^)	right	2591 ±123	2560 ±97
VO_2_max	(l min^-1^)		3.79 ±0.19	3.52 ±0.13
VO_2_max / body weight	(l min^-1^ kg^-1^)		50.2 ±2.3	40.2 ±2.0**
Maximal heart rate	(1 min^-1^)		188.2 ±3.9	176.2 ±3.3*
Femoral art. lumen diameter	(mm)	right	9.01 ±0.23	9.49 ±0.23
Calf blood flow at rest	(ml 100ml^-1^ min^-1^)	left	2.16 ±0.42	2.0 ±0.25
		right	2.38 ±0.53	1.9 ±0.20
Calf art. flow reserve	(ml 100ml^-1^ min^-1^)	left	14.9 ±2.0	17.9 ±1.9
		right	15.3 ±2.5	17.5 ±1.4

Mean ±SE

* for p<0.05

** for p<0.01

***for p<0.001 unpaired t-test

IMT = Intima-media thickness, PT = Peak torque, VO_2_max = maximal oxygen uptake per time.

Among the traditional blood cardiovascular risk factors, fasted total cholesterol and LDL were found to be significantly higher (35% and 46%) in MA compared to YG, however, well within the normal range and even below the average of the untreated German male population aged 35–65 years i.e. 236 (±SD of 46) mg/dl (or 234 mg/dl for the age range 40–79 years) and 168 (±SD of 43) mg/dl, respectively [56, 57].

Expectedly, this is quite obviously at variance to the suggested pharmacological target ‘reference’ values for primary prevention without further risks in non-smokers i.e. <200 mg 100ml^-1^and <160 mg 100ml^-1^, respectively.

Homocysteine, as a non-traditional age-related cardiovascular risk factor [54], was virtually identical between the MA and YG. While fasted blood levels of glucose were not different between groups, HbA1c was about 5% higher in MA, however, also well within the normal range. In line with these marginal differences in blood parameters, the bilateral carotid IMT, as more integral marker and endpoint of cardiovascular risk, was normal but about 22% and 18% higher on left and right side, respectively, in MA compared to YG. Leg vascular (functional) parameters at rest were similar in MA and YG with regard to femoral artery lumen diameter, resting calf blood flow, and calf arterial flow reserve (post-occlusion vasodilatative capacity). Also, these parameters were similar in the left and right leg, which may additionally support the notion of vascular health in both, MA and YG.

### Comparability of vastus lateralis muscle histomorphometry between MA and YG

Among the histomorphometrical parameters (**Table 2 ‘Histomorphometry of vastus lateralis muscle capillarisation and fiber composition’**) determined in the VLat muscle biopsies, we found that capillary density analysed per rectangular area (as well as per total fiber area within the rectangular area), the capillary to fiber ratio and the capillary contacts per fibers in total or per each of the type 1, 2a or 2x were similar between MA and YG. This also applied to the calculated ratio of capillary contacts per unit fiber area for all fibers as well as for each fiber type 1, 2a or 2x. The fiber composition in terms of percentage of fiber type 1, 2a and 2x count was similar as well between MA and YG.

**Table 2:** Histomorphometry of vastus lateralis muscle capillarisation and fiber composition.

		Young (YG)	Middle-aged (MA)
N		10	13
Fibers analysed		156 ±10	135 ±9
Capillary density (count / area)	(mm^-2^)	295 ±30	285 ±16
Capillary count / total fiber area	(mm^-2^)	354 ±33	330 ±17
Capillary to fiber ratio	(ratio)	2.18 ±0.16	2.20 ±0.13
Capillary contacts—all fibers	(count/fiber)	4.87 ±0.31	4.90 ±0.23
Capillary contacts—fiber type 1	(count/fiber)	5.00 ±0.31	5.20 ±0.27
Capillary contacts—fiber type 2a	(count/fiber)	5.01 ±0.38	4.75 ±0.33
Capillary contacts—fiber type 2x	(count/fiber)	4.68 ±0.30	3.74 ±0.38
Capillary contacts / area—all fibers	(n/10^3^μm^3^)	.818 ±.099	.747 ±.048
Capillary contacts / area—fiber type 1	(n/10^3^μm^3^)	.896 ±.103	.797 ±.045
Capillary contacts / area—fiber type 2a	(n/10^3^μm^3^)	.802 ±.070	.677 ±.065
Capillary contacts / area—fiber type 2x	(n/10^3^μm^3^)	.784 ±.103	.641 ±.071
Percentage fiber type 1	(%)	43.2 ±4.3	41.9 ±4.1
Percentage fiber type 2a	(%)	39.5 ±5.5	34.7 ±4.8
Percentage fiber type 2x	(%)	21.2 ±3.9	23.6 ±3.3

Mean ±SE

* for p<0.05

** for p<0.01

***for p<0.001 unpaired t-test

No significant differences detected between MA and YG for any of the parameters presented.

### Differences in microvascular CEUS parameters and leg blood flow/ conductance at rest, during and after exercise between MA and YG

#### Resting conditions

**Fig 5:**
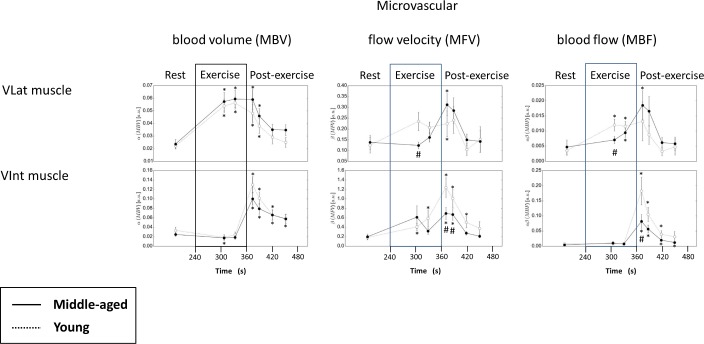
‘Microvascular blood volume (MBV), flow velocity (MFV) and blood flow (MBF)’. Mean (±SEM) microvascular blood volume (MBV, *left*), flow velocity (MFV, *middle*), and blood flow (MBF, *right*) in the vastus lateralis (VLat; *upper panel*) and the vastus intemedius (VInt; *lower panel*) muscle ROI of middle-aged (MA, n = 15) compared to young (YG, n = 11) at rest (two measurements), after 70 and 95 s of isometric exercise and 15, 30 60 and 90 s post-exercise. Note that these data were individually calculated from individual RC curve regression before averaging them for MA or YG (for mean RCs and regression lines per group see Fig 4A). # for p<0.05 by unpaired Student’s t-test middle-aged MA vs. YG. * for p<0.05, ** for p<0.01, and *** for p<0.001 by paired Student’s t-test for changes relative to rest (basline) within the group of MA or YG.

As shown in Fig 3, the initial 180 s of constant antecubital Sonovue infusion at rest led to an increase (with circulatory delay) of background-subtracted mean Sonovue microbubble signal, i.e. acoustic intensity (AI, reflecting microbubble tissue concentration) that reached equilibration within both, the VLat (*upper panel*) and VInt (*lower panel*) muscle ROIs without significant differences between MA and YG. On the base of such equilibrated resting conditions, two consecutive 25-s-RC were recorded (190 s and 240 s) after high-MI microbubble destruction at 190 s and 215 s (see Fig 1A). These RC are presented as means for the group of MA and of YG in Fig 4A (*left panel*) together with each group’s means of the individually calculated regression curves in Fig 4B (*left panel*). The individual regression lines were used for calculations of individual resting values of MBV, MFV and MBF according to the model equation (see Methods) which are presented as means for MA and YG (Fig 5) without significant differences. (See also **‘S1 Video. Example of a CEUS recording at rest, during and after isometric contraction**’).

**Fig 6:**
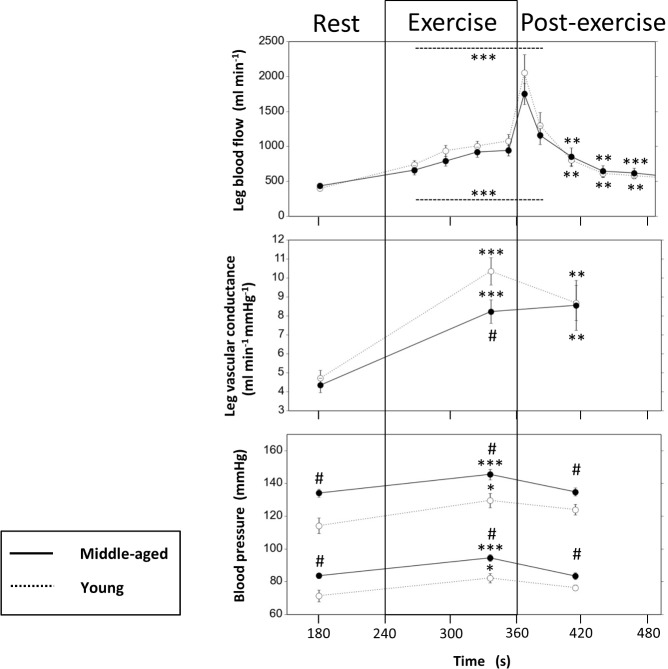
‘Total leg blood flow and conductance’. Mean (±SEM) total leg blood flow (femoral artery Duplex-Doppler flow), calculated total leg vascular conductance (leg blood flow per mean arterial pressure), and systolic as well as diastolic brachial arterial blood pressure at rest in middle-aged (MA, n = 15) compared to young (YG, n = 11) males after ~90 s of isometric exercise and ~60 s post-exercise. # for p<0.05 by unpaired Student’s t-test middle-aged MA vs. YG. * for p<0.05, ** for p<0.01, and *** for p<0.001 by paired Student’s t-test for changes relative to rest (baseline) within the group of MA or YG.

Femoral artery Doppler at rest showed similar total leg blood flow in the range of 400 ml min^-1^ as well as similar leg conductance between MA and YG despite significantly slightly higher systolic and diastolic blood pressure levels in MA (Fig 6). Notably, these resting (immediately pre-exercise) values are slightly different from the initial blood pressure values measured at initial screening (Table 1).

#### Isometric exercise

As shown in Fig 3 (*middle panel*), the start of isometric knee extensor contraction prompted an immediate reduction in AI in both, the VLat and VInt muscle relative to equilibrated levels reached at rest (Fig 3, *left panel*, 160–180 s). In the VInt muscle, this reduction in AI remained about constant and comparable between MA and YG for the initial 70 s observation period without high-MI microbubble destruction. However, in contrast, the VLat showed a slow progressive increase in AI, which transiently reached significantly lower values in MA than in YG between 260 s and 270 s of the protocol (Fig 3, *middle panel*) reflecting a transiently delayed increase in microbubble density or AI in MA vs. YG during early isometric contraction. Moreover, the analyses of the intra-exercise RC curves obtained after microbubble destruction at 310 s and 335 s of the protocol (Fig 4A, mean measured RC; Fig 4B, mean regression lines of MA and YG) revealed, that ongoing contraction of VLat muscle induced a (by then) similarly large MBV increase (Fig 5, *left panel*), which, however, was associated with a significantly lower MFV (Fig 5, *middle panel*) and accordingly a significantly lower MBF (Fig 5, *right panel*) in MA compared to YG. In the VInt muscle, however, MA and YG showed similar contraction-induced MBV decreases (Fig 5, *left panel*, as already indicated by AI in Fig 3, *middle panel*) associated with similar moderate MFV increases (Fig 5, *middle panel*), which together yielded no exercise-induced MBF increases at all and, accordingly, no differences between MA and YG (Fig 5, *right panel*). (See also **‘S1 Video. Example of a CEUS recording at rest, during and after isometric contraction’**).

Importantly, the lower MBF in contracting VLat muscle of MA compared to YG was not reflected in the total leg blood flow as assessed as femoral artery Doppler (Fig 6), though Doppler demonstrated a significant about two-fold leg blood flow increase after 1 min of contraction in both groups. However, calculated leg vascular conductance (Fig 6), i.e. normalizing for (the significantly higher) mean arterial pressure (not shown) revealed significantly attenuated vasodilation with contraction in MA compared to YG.

#### Post-exercise hyperemia

Upon exercise cessation, the release of intramuscular pressure allowed for post-exercise hyperemia i.e. an immediate increase in AI between 365 and 380 s, which was several-fold higher in the VInt (i.e. the region with more vascular compression) than in the VLat muscle and, importantly, was significantly lower (or delayed) in MA compared YG (Fig 3, *right panel*). When analysing post-exercise RC curves within VInt (Fig 4A, *right panel*) by regression (Fig 4B, *right panels*), MBV tended to be and MFV in fact was significantly lower within the first 60 s after exercise cessation resulting in an about 50% lower MBF in MA compared to YG within the first 30 s (Fig 5). Notably, the post-exercise MBF in the VLat muscle, in contrast, reached only about 30% and <10% of VInt muscle MBF in MA and YG, respectively, with no significant difference between the groups (Fig 5, *right panel*). Thereby, the hyperemia, i.e. the increase of MBF from exercise to post-exercise conditions, was small (likely compensating for lower intra-exercise MBF) in MA and absent YG within their VLat muscle, while, notably, it was found to be about ten-fold in their VInt (Fig 5, *middle and right panel*). (See also **‘S1 Video. Example of a CEUS recording at rest, during and after isometric contraction’**).

Total leg blood flow as assessed by Doppler (Fig 6) showed a post-exercise peak (mean values between 1500 and 2000 ml min^-1^) that coincided well with the post-exercise MBF maxima in CEUS recordings. However, it failed to reflect the significant and massive MBF difference detected by CEUS in the VInt between the MA and YG. Likewise, also calculated leg conductance (Fig 6) did not detect the differences in MBF, at least after 60 s post exercise. It should be noted, that large regional differences in the MBF response like between the VLat and VInt muscle during and post-isometric exercise as well as region-specific differences between MA and YG may only affect a relatively small portion of the total leg blood flow (increase). Therefore, the Doppler flow may only by trend or (in terms of conductance) partly reflect MBF in one specific muscle region, if other regions receive little flow like the VInt muscle during contraction.

A video example **(‘S1 Video. Example of a CEUS recording at rest, during and after isometric contraction’)** shows a representative CEUS recording during resting conditions, isometric exercise, and post-exercise hyperemia in both, the VLat and VInt muscle (For details see legend).

Because the regression lines (Fig 4B) as the data base for individual MBV, MFV and MBF calculation were presently obtained by modifying the common (so far published) approach [33, 50] in order to improve RC origin determination, we compared the two methods (see ‘Methods‘ section, ‘CEUS calculations’), a) (‘new’) and b) ‘conventional’) for calculating the loss of microbubble signal (AI) with high-MI destruction, i.e. the difference between pre-destruction AI and AI at RC-origin determined, which was expressed as percentage of the overall background-subtracted pre-destruction microbubble AI (**‘S1 Fig. Methodology of CEUS replenishment curve calculation’):** Briefly, these two (‘new’ or ‘conventional’) calculations of the loss in microbubble signal indicate how well microbubble high-MI-destruction approaches the microbubble-free background i.e. an ideal 100% loss the actually pre-existing microbubble signal.Obviously, the present method a) (**see S1B Fig, ‘new’ in ‘S1 Fig. Methodology of CEUS replenishment curve calculation’**), i.e. extrapolation of the regression line of the RC data between 1 s and 25 s to time 0 s (i.e. fixed assumption on x only but not y of the RC origin,) yielded close to 100% microbubble destruction irrespective of muscle region or condition (rest/exercise). This was not the case with the so far published method b) (**see S1B Fig, ‘conventional’ in ‘S1 Fig. Methodology of CEUS replenishment curve calculation’**) which averages the apparative signal of the first 0.5 s after destruction (fixed assumption on both, y and x of the RC origin).

## Discussion

Aging at clinical health has been found to be associated with attenuated limb blood flow responses to (dynamic) exercise, which in humans is consistent across the studies at least for the leg [1–5, 10, 17, 20]. Except for an age-related capillary rarefaction [22, 23], which is likely to decrease the recruitable/available MVU capillary network size, the alterations in microvascular function underlying age-related impairments of blood supply to exercising muscle, i.e. local O_2_-supply, are largely unknown in humans [3]. Moreover, blood flow studies with regard to aging have mostly involved dynamical type of exercise, however, performance in elderly may also largely depend on isometric exercise tolerance, i.e. on local endurance as frequently required for postural control and locomotion involving large muscle mass.

Therefore, we presently used CEUS to compare microvascular functional parameters of resting and isometrically contracting VLat and VInt muscle between fasted MA and YG males and controlled for VLat histomorphology of capillarsation. CEUS has been shown to detect both, insulin/feeding- and exercise-induced microvascular responses in humans and mammals in terms of increases in MBV i.e. microbubble density within a tissue ROI (interpreted as ‘capillary recruitment’ by most CEUS users, but likely a complex increase in exchange surface, as discussed below) as well as increases in MBF i.e. the product of MBV * MFV, even at unchanged total limb blood flow [25, 31–33, 39, 49, 50].

As a main finding, MA compared to YG reveal a significantly initial delay in MBV increase (AI increase due to microbubbles) in VLat upon early isometric contraction (Fig 3, *middle panel*) and, furthermore (with ongoing contraction and then aligned AI between MA and YG), a significantly lower (calculated) MFV and consecutively a lower MBF (Fig 5, *middle panel*). Whether such age-related impaired skeletal muscle microcirculation as determined by CEUS actually implies a limitation in local rbc or O_2_ supply and endurance during light isometric contraction, has to remain open at present (see limitations of the study below).

Of note, there were slightly, non-significantly higher levels of post-exercise MBV, MFV, and MBF in MA compared to YG, which are presently regarded as compensatory (for lower MBF during previous contraction) within a rather small hyperemic response compared to that observed in VInt.

Regarding the VInt muscle, no such difference was detectable during isometric contraction, since MBV (Fig 2, *middle panel*; Fig 5, *left panel*) did not increase but, in contrast, was reduced most likely through high intramuscular pressure in both, MA and YG (Fig 5, *right panel*) despite some MFV increases (Fig 5, *middle panel*). However, exercise cessation prompted a massive reactive (post-occlusive) hyperemia (several-fold higher than in the VLat muscle), which, importantly, was significantly delayed in MA vs. YG in terms of AI increase within the first 15 s after end of contraction (Fig 4, *right panel*) and, thereafter, significantly attenuated in MA vs. YG in terms of MBV (Fig 5, *left panel*, non-significant trend), MFV and MBF as calculated from RCs (Fig 5, *middle and right panel*). This may suggest an impaired microvascular functional capacity in MA vs. YG in the VInt, which upon release of intramuscular (vessel-occluding) pressure limits post-exercise hyperemia in MA vs. YG.

Our present data therefore indicate, that as early as from the 5^th^ decade of males, relevant age-related changes in microvascular exercise responses are detectable with CEUS.

Whether this implies limitations O_2_-delivery or local muscle performance warrants further studies. These findings of a compromised microcirculation early upon exercise are in line with data from the trapezius muscle of rats [8] showing lower microvascular O_2_-partial pressure at the onset of contraction, potentially contributing to O_2_ deficit and premature fatigue.

Importantly, these intra- or post-exercise microvascular differences between MA and YG were not reflected in femoral artery Doppler recordings of total leg blood flow and could only be detected by calculated vascular conductance during but not after exercise. Possibly, the post-exercise MBF peak in the VInt muscle was to short and occurred in a too small tissue portion to be reflected by total leg conductance. Because previous exercise blood flow studies on healthy human aging used total limb (sectional) blood flow, but not intramuscular microvascular analyses, they might have failed to detect the rather early onset of age-related impairments [16] and/or reported negative findings, e.g. in the forearm [3, 9, 10]. Notably, the age-related loss in muscle mass, starting from the age around 40 years as well and reaching about 10% at 50 years [13, 14], are suggested to involve microcirculatory among other limitations [15]. However, as there may be no simple cause-effect-relationship between local and systemic factors of nutritional blood flow, functional capacity, and muscle mass, the role of early microvascular dysfunction in sarcopenia remains to be assessed.

In humans, age-related impairments in microvascular (endothelial) function, have recently been established for another very important stimulus, i.e. insulin or feeding, which may change or redistribute microvascular flow and enhance capillary exchange surface with or without detectable changes in total limb blood flow [33, 39, 58]. Exercise may resemble insulin as a physiologic stimulus for endothelial NO-dependent acute and recruitment of skeletal muscle capillary fiber-adjacent exchange surface but, in addition, it increases total muscle perfusion and conductance as presently seen due to multiple other non-mechanical vasodilatative mechanism that ascend to feeding and conduit arteries [3, 25, 39, 59]. While human data on age-related microvascular exercise response are lacking, one recent study has shown an age-related attenuation in human CEUS microvascular exercise response after 60 min after dynamic exercise [60] which, however, was under conditions of feeding and age-related differences in plasma insulin levels (together with CEUS differences at baseline rest). Even more important, these data obtained in a group aged around 67 years could easily reflect lower capillary density as an important age-related factor, which commonly has not been controlled for in most, if not all, recent CEUS studies on the insulin effect alone [3, 33, 39].

The present CEUS study, therefore, investigated capillary histomorphometry at the exact site of CEUS measurement, including capillary density, fiber type-specific capillary contacts, and their supplied fiber area as well as fiber composition. This is important, because differences in MBV as detected by CEUS may theoretically reflect structural differences in capillary network, i.e. capillary density or recruitable capillary exchange surface/volume of a MVU and, therefore, conclusion on microvascular dysfunction may be erroneous, especially because the exact MVU anatomy and function in humans is not known [25]. While, starting from the age of 40 years, there is an ongoing loss of muscle mass or fiber area (which should geometrically increase capillary density), aging without training countermeasure clearly reduces capillary contacts themselves along with VEGF expression [22, 23] though at preserved angiogenetic exercise response [24]. Furthermore, the metabolic syndrome, as an age-immanent factor itself, appears to contribute to capillary rarefaction [26, 47].

We presently demonstrate, however, that the findings on calculated MBV or reached AI (before microbubble destruction) with CEUS cannot be attributed to a significant, age-related difference in capillary density, capillary to fiber ratio, or any overall or fiber type-specific difference in capillary contacts or supplied fiber area per contact. Thus, there obviously is rather a *functional* than a *structural* impairment in microcirculation in healthy MA vs. YG males, even under the noteworthy conditions of the present study that muscle mass, muscle function (of quadriceps muscle) and arterial reserve (of the calf muscles) appear to be preserved.

Our age-related findings may therefore represent attenuation of different exercise-responsive vasodilative mechanisms (e.g. O_2_- or NO-dependent), which in view of similar total leg blood flow, may be located in high order arterioles (precapillary sphincters) controlling distribution, while in case of differences in leg blood flow feed arteries and low order resistance arterioles may be additionally involved. Sinkler and Segal [30] demonstrated in mice gluteus maximus muscle that aging may attenuate the vasomotion O_2_-response consistently along the total resistance network, however, age-effects on (endothelium dependent) acetylcholine responses were more complex: While in feed arteries it was attenuated with age—reflecting the findings on total limb post-occlusion or acetylcholine responses in humans—more distal arterioles were, in contrast, more sensitive with higher age. Moreover, resting spontaneous vasomotor tone as well α-adrenergic vasoconstriction differed with age and microvascular branches. How such differential findings along arterial branching translate to human skeletal muscle microvasculature with still largely unknown 3D-anatomy remains an open field of research, especially with regard to exercise type, intensity, duration, involved fiber composition, training status and other factors [8, 25].

However, since, starting from the 5^th^ decade in men, endothelium- (NO-) dependent vasodilation with acetylcholine, after arterial occlusion or upon insulin/feeding are clearly attenuated with age, the metabolic syndrome or type-2-diabetes [3, 25, 28, 39], NO-dependent mechanism are likely to, at least in part, play a role in the presently shown age-related microvascular dysfunction during exercise in middle-aged males. Beside endothelial eNOS activity or NO metabolism, limitations of NO-availability may possibly also arise neuronal NOS as another NO sources, that shows an age-related impact on microcirculation [61]. Furthermore, aging may lead to a NO-dependent redistribution of microvascular blood flow distribution from ‘red’ and ‘white’ muscle groups/ regions with consequences for endurance training effects [8, 62, 63]. Though these data may not be easily extrapolated from rat to men, it is important to note, that the present biopsy data from the actual CEUS site exclude differences in fiber composition or in fiber type specific capillary contacts or capillary supply area between MA and YG.

As a note of caution regarding limitations of the CEUS method, it should be stated, that it is presently unclear, to what extent the Sonovue (SF_6_) microbubbles with a diameter of ~2μm reflect the flux of rbc, which in the capillary network may be differential between neighboring or even within the same capillary upon transition from rest to exercise [64, 65]. It is likely, that microbubbles behave more like platelets (1.5–3 μm diameter) or plasma. Moreover, it is unclear and a matter of debate whether an increase in microbubble density, as reflected by increased MBV, is mainly due to an actual de-novo recruitment of previously non-flowing capillaries (‘traditional’ view) or, more likely, to several other factors leading to recruitment of exchange area like alterations in rbc or plasma flow (distribution), capillary volume, longitudinal recruitment or in endothelial surface layers, all occurring in capillaries of which >80% receive rbc or plasma flux already at rest, at least in mammals (64, 65). However, whatever the factors of increased microbubble density within the tissue are, our findings on calculated MBF (which is e.g. lower in contracting VLat of MA compared to YG) should reflect blood (plasma) supply per time and tissue cross-sectional area. The exercise-induced increase in microbubble velocity (which was lower in VLat of MA compared to YG) appears to be compatible with the novel concept of exercise-induced recruitment of exchange surface in skeletal muscle [65]. However, conclusion regarding the actual rbc or O_2_-supply to working muscle fibers should presently be avoided, because the rbc flux is not known and, as important, because the relation between microcirculation and the pattern and distribution fiber (type) i.e. motor unit recruitment is totally unclear in the present study. Given these uncertainties, it is noteworthy, that, to our knowledge, this is the first human CEUS study to control for capillary histomorphometry including fiber-type-specific contacts at CEUS site as a previously neglected important structural factor of differences in CEUS findings. Our biopsy histomorphometry shows, that the ROI analysed within the Vlat covered about 70.000 capillaries, which should ‘average’ possible inhomogeneities in rbc or plasma flux between neighboring capillaries as observed by intra-vital microscopy.

Furthermore, comparability of groups within a cross-sectional aging study requires control of multiple vascular risk factors, that depend on aging and the aging-immanent metabolic syndrome and may generally affect structural or functional endpoints of clinical vascular studies, such as capillarisation, arterial stiffness or endothelial function in in response to arterial-occlusion, insulin or exercise stimuli [25, 28, 35, 37, 39, 41, 42, 48, 53]. We presently aimed to match an only moderately aged group (MA ~44 years) to that of YG (~24 years) for mass and maximal strength of quadriceps as well as of forearm muscles and furthermore for absolute VO_2_max as an index of integral cardiorespiratory function/fitness. This approach may have, somewhat inevitably, lead to the differences in body weight and BMI (26.9 vs. 23.1 kg m^-2^ in MA and YG, respectively) which should mainly be attributable to higher absolute and percentage body fat (though these parameters were not assessed). Thus, despite a 10 kg higher body weight (i.e. a weight gain of 0.5 kg per year of aging) VO_2_max per quadriceps muscle mass (not shown) was not significantly different. As an alternative approach, matching for BMI or for VO_2_max per body weight (which was significantly lower in MA vs. YG) would, however, likely have resulted in lower muscle mass or strength in MA, lower absolute VO_2_max, and potentially reduced capillary density–all of which are rarely analysed in studies matching for BMI, i.e. for a parameter that does not distinguish between fat and muscle. On the other hand, our present muscle- instead of BMI-related matching may have contributed to significantly higher, though still normal levels in diastolic blood pressure, total cholesterol, LDL and HBA1c, while other critical factors of endothelial function like triglycerides, HDL, and homocysteine remained similar. On this background of cardiovascular risk profiles, there was a significantly greater carotid IMT in MA vs. YG (again well within the normal range). Beside comparability in capillarisation and fiber morphology at the VLat CEUS site, the presently studied MA and YG groups had similar calf maximal vascular post-occlusion hyperemia. Furthermore, all blood flow parameters obtained at rest including femoral artery Doppler, bilateral calf blood flow (venous occlusion plethysmography), and CEUS parameters were very similar between MA and YG. Control for muscle mass may have been crucial to obtain comparability in resting (baseline) leg blood flow between MA and YG, as pointed out by Donato et al. [4].

Taken together, we consider the present markers of a ‘beginning metabolic syndrome’, as an age-characteristic difference ‘at health’, because they were within the normal range and did not significantly impact capillarisation, muscle mass, VO_2_max, leg or calf blood flow parameters or glucose [26, 48].

## Conclusions

In summary, this is the first CEUS study in humans to demonstrate a rather early effect of healthy aging on the microvascular responses to isometric exercise while controlling for capillary histomorphology and fiber composition at the actual site of CEUS measurements. We demonstrate age-related *functional* rather than *structural* impairments in microvascular blood flow during light prolonged isometric contraction in areas with increased blood flow as well as during post-exercise hyperemia in previously flow-occluded muscle areas. It remains to be assessed, whether this finding may imply impaired local O_2_-supply or even local isometric endurance which is critically involved in postural or limb stabilisation as required for locomotion and skilled movements. The present experimental protocol may enable a well-tolerable standardized clinical model to evaluate skeletal muscle microvascular function even at an older age and in various metabolic, muscle, cardiorespiratory or other diseases.

## Supporting information

S1 Dataset Individual parameters obtained from CEUS replenishment curves. (XLS)

S2 Dataset Individual values underlying Tables and Fig 6. (XLSX)

S1 Video Example of a CEUS recording at rest, during and after isometric contraction’. The video simultaneously shows three separate consecutive low-MI-CEUS recording of Sonovue microbubble replenishment in one middle-aged subject demonstrating the conditions of rest (*left*), exercise (*middle*), and 15 s post-exercise (*right*), with each video covering the first 15 s after high-MI microbubble destruction, i.e. the interval with the major and most rapid portion of microbubble replenishment. Thereby, the *upper panel* shows the real-time measurements of Sonovue replenishment (i.e. appearing and disappearing microbubbles) whereas the *lower panel* demonstrates the identical recording interval in terms of corresponding cumulative CEUS signal over time. Each recording represents the muscle area scanned by the linear parallel 9D-L scanner including the vastus lateralis (*above*) and intermedius (*below*) muscle, separated by the intermuscular septum (with minor position changes through contraction). The scanner was aligned to muscle fiber orientation (from left to right). Note that microbubble distribution in the vastus intermedius (VInt) muscle is non-homogenous at rest (*left*, *lower panel*), however, much more homogeneous with post-exercise hyperemia (*right*, *lower panel*), whereas exercise (isometric contraction) effectively inhibits microvascular blood flow (*right*, *middle panel*). In contrast, in the vastus lateralis (VLat) muscle, exercise moderately increases and homogenizes the distribution of the CEUS signal from insonated Sonovue microbubbles.
(MOV)

S1 Fig Methodology of CEUS replenishment curve calculation’. Representative example for the presently used new method (see S1a Fig) ‘new’) compared to the so far published (see S1a Fig, ‘conventional’) method for determination of the replenishment curve (RC) origin after high-MI Sonovue microbubble destruction at rest and under exercise conditions. An ideal 100% high-MI-flash microbubble destruction would decrease acoustic intensity (AI) from the upper margin (pre-destruction AI level) to the lower margin (background AI level before microbubble arrival) of the grey shaded area.
According to the ‘conventional’ approach, the RC curve origin is set at ~0.28 s after the high-MI impulse and to the signal (AI) average of the initial 0.5 s to calculate the regression line. In contrast, the present ‘new’ method uses least-square-regression of RC from 1 s to 25 s post high MI-flash to determine RC curve origin at high-MI flash end (0 s post- flash) by extrapolation. This ‘new’ approach yields an RC origin that is closer to measured microbubble-free background (lower margin of the shaded rectangle) than with the ‘conventional’ approach, which obviously determines RC origin considerably above this microbubble-free background (lower margin of the shaded rectangle), i.e. it underestimates the percentage microbubble destruction (reduction in total microbubble signal) with the high-MI flash.
S1b Fig compares the percentage loss of microbubble signal (microbubble high-MI destruction by boxplots for all subjects under test at rest (left), during exercise (middle), and post-exercise (right) in the vastus lateralis (VLat; *upper panel*) and in the vastus intermedius (VInt; *lower panel*) muscle. Obviously, the deviation from 100% microbubble destruction (signal loss) is much smaller with the present ‘new’ approach as compared to the ‘conventional’ approach, which appears to be subjected to the largest error, when the microvascular blood volume (MBV) and flow (MBF) is minimal, like e.g. during contraction of the VInt muscle.
(TIF)
