# Supplementary figures and images for: Age-related differences in skeletal muscle microvascular response to exercise as detected by contrast-enhanced ultrasound (CEUS)
Source: PLoS One. 2017 Mar 8;12(3):e0172771. doi: 10.1371/journal.pone.0172771 (PMC5342194; doi:10.1371/journal.pone.0172771)

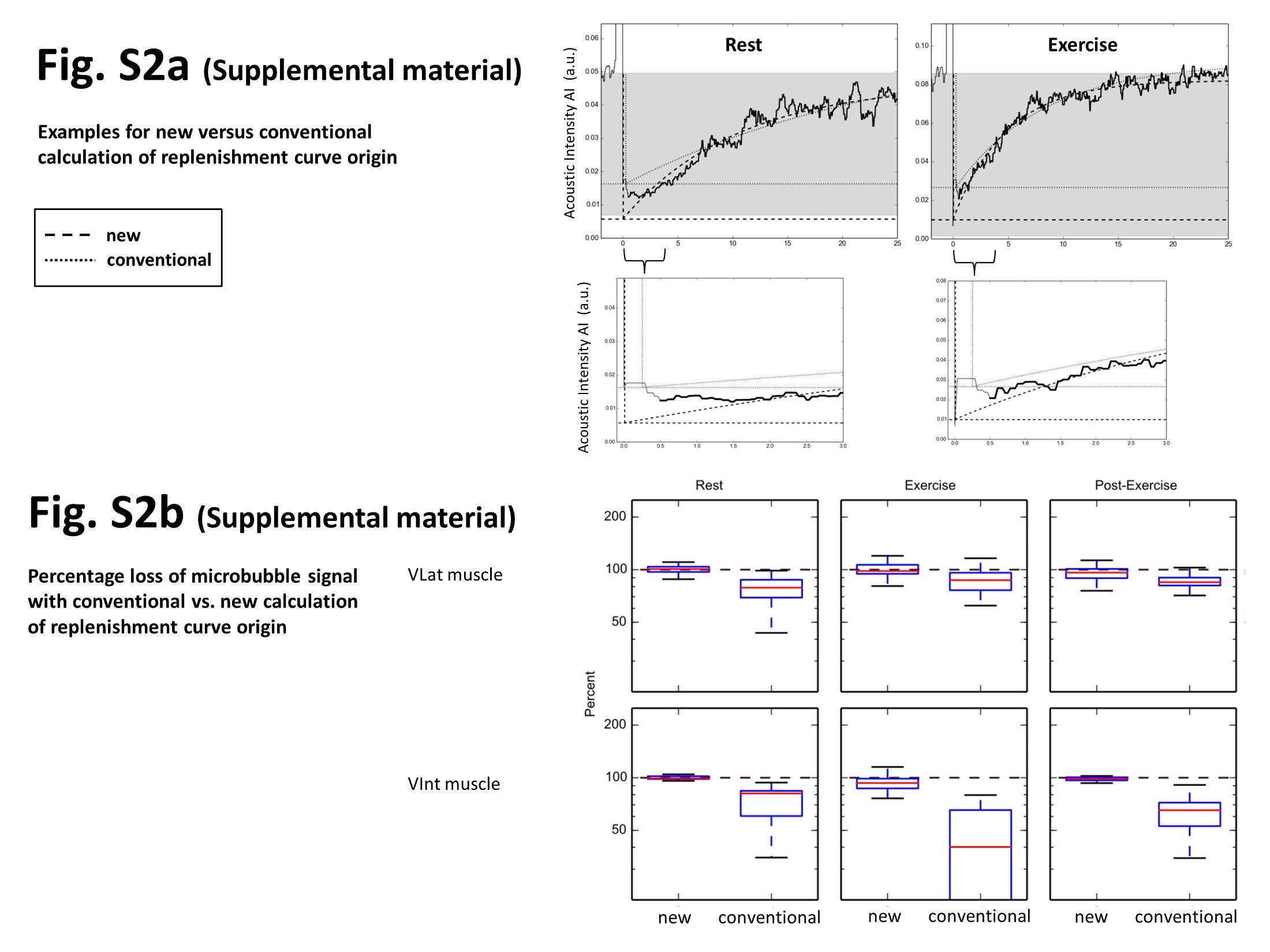

Supplement: S1 Fig — Representative example for the presently used new method (see S1a Fig) ‘new’) compared to the so far published (see S1a Fig, ‘conventional’) method for determination of the replenishment curve (RC) origin after high-MI Sonovue microbubble destruction at rest and under exercise conditions. An ideal 100% high-MI-flash microbubble destruction would decrease acoustic intensity (AI) from the upper margin (pre-destruction AI level) to the lower margin (background AI level before microbubble arrival) of the grey shaded area. According to the ‘conventional’ approach, the RC curve origin is set at ~0.28 s after the high-MI impulse and to the signal (AI) average of the initial 0.5 s to calculate the regression line. In contrast, the present ‘new’ method uses least-square-regression of RC from 1 s to 25 s post high MI-flash to determine RC curve origin at high-MI flash end (0 s post- flash) by extrapolation. This ‘new’ approach yields an RC origin that is closer to measured microbubble-free background (lower margin of the shaded rectangle) than with the ‘conventional’ approach, which obviously determines RC origin considerably above this microbubble-free background (lower margin of the shaded rectangle), i.e. it underestimates the percentage microbubble destruction (reduction in total microbubble signal) with the high-MI flash. S1b Fig compares the percentage loss of microbubble signal (microbubble high-MI destruction by boxplots for all subjects under test at rest (left), during exercise (middle), and post-exercise (right) in the vastus lateralis (VLat; upper panel) and in the vastus intermedius (VInt; lower panel) muscle. Obviously, the deviation from 100% microbubble destruction (signal loss) is much smaller with the present ‘new’ approach as compared to the ‘conventional’ approach, which appears to be subjected to the largest error, when the microvascular blood volume (MBV) and flow (MBF) is minimal, like e.g. during contraction of the VInt muscle. (TIF) [file pone.0172771.s004.tif]
